# Detection and follow-up of chronic health conditions in Rio de Janeiro – the impact of residency training in family medicine

**DOI:** 10.1186/s12875-021-01542-5

**Published:** 2021-11-13

**Authors:** Adelson Guaraci Jantsch, Bo Burström, Gunnar Nilsson, Antônio Ponce de Leon

**Affiliations:** 1 Instituto de Medicina Social, Rio de Janeiro State University, Bloco D e E, R. São Francisco Xavier, 524 – 7th floor - Maracanã, Rio de Janeiro, RJ 20550-900 Brazil; 2 Department of Global Public Health at the Karolinska Institute, Solna, Sweden; 3 Department of Neurobiology, Care Sciences and Society at the Karolinska Institute, Solna, Sweden

**Keywords:** Primary Health Care, Family Practice, Health Workforce, Developing Countries

## Abstract

**Abstract:**

**Background:**

There is a need for evidence that residency training in family medicine can benefit the care of patients in primary care in low- and middle-income countries. We tested the hypothesis that two years of residency training in family medicine enables doctors to better detect chronic health conditions while requesting fewer laboratory tests and providing more follow-up visits.

**Methods:**

We performed a retrospective longitudinal observational analysis of medical consultations from 2013 to 2018 in primary care in Rio de Janeiro, comparing doctors without residency training in family medicine (Generalists) versus family physicians (FPs). Multivariate multilevel binomial regression models estimated the risks of patients being diagnosed for a list of 31 chronic health conditions, having a follow-up visit for these conditions, and having laboratory tests ordered from a list of 30 exams.

**Results:**

569.289 patients had 2.908.864 medical consultations performed by 734 generalists and 231 FPs. Patients seen by FPs were at a higher risk of being detected for most of the chronic health conditions, at a lower risk of having any of the 30 laboratory tests requested, and at a higher risk of having a follow-up visit in primary care.

**Conclusions:**

Residency training in family medicine can make physicians more skilled to work in primary care. Policymakers must prioritize investments in capacity building of healthcare workforce to make primary care truly comprehensive.

## Introduction

Capacity building of human resources for primary health care (PHC) is a sensitive issue in global health [1]. For many low- and middle-income countries [2] (LMIC) the inability to train health care providers is a critical structural problem [3, 4]. Family medicine (FM) is a key element that adds comprehensiveness to PHC [5, 6]. However, Residency Training in FM (RTFM) – the gold-standard for post-graduate training of medical health care providers in PHC – is usually not available or, at most, is undervalued and incipient in LMIC. Furthermore, the idea that PHC can easily be performed by any physician without specialized training is a common misconception among many policymakers in countries where selective PHC is the rule [7].

Family physicians (FPs) are trained to provide comprehensive patient-centered care [8, 9], managing the most prevalent health conditions in the community, and making good use of the resources available [10, 11]. In high-income countries (HIC) with strong PHC, FM is a well-established medical specialty and FPs comprise the majority of the medical workforce [12]. Nevertheless, leaders in FM and primary care providers routinely advocate for improvements [13], showing that strong PHC leads to better performance on health indicators while spending less money [14, 15]. Studies from LMIC showing the RTFM’s impact on the quality of care may encourage policymakers to invest in capacity building of human resources for PHC [16–18].

Being able to diagnose prevalent chronic health conditions (CHCs), making rational use of laboratory tests and provide follow-up consultations are necessary competencies that any physician working in PHC must have [10, 19, 20]. This study tests the hypothesis that RTFM enables FPs to be more competent to detect CHC while requesting fewer laboratory exams and providing more follow-up visits for patients with CHC. It aims specifically to estimate the risks of patients (1) being diagnosed for a CHC; (2) having a laboratory test (LT) requested in one medical consultation; and (3) having a follow-up visit in PHC, according to the type of physician, i.e., FPs or physicians without RTFM (Generalists). Finally, (4) we aimed to determine the Population Attributable Fractions and estimate what would be the change in the amount of LTs requested and incident cases of CHC per year if all medical consultations were carried out by FPs.

## Methods

### Study design and data source

This is a retrospective longitudinal observational analysis of medical consultations performed by FPs and Generalists in the public PHC system in the Rio de Janeiro municipality from January 2013 to December 2018. Each medical consultation (the unit of analysis) was considered as a binary event (diagnosed versus non-diagnosed, LT requested versus LT not requested, follow-up consultation versus non-follow up consultation).

Information from 965 physicians and 999.364 patients registered in one health district were all anonymized and the Rio de Janeiro Health Department (responsible for the safekeeping of the data) provided the consent to use it in research. The study was conducted in accordance with the 466/12 resolution from the Brazilian National Health Council [21] and the Declaration of Helsinki and it was approved by the Rio de Janeiro Municipal Health Department (RJ-MHD) research ethics board. It is registered under the number 03795118.0.0000.5279.

### Setting

The Family Health Strategy (Estratégia de Saúde da Família – FHS) is the Brazilian federal policy for public PHC launched in 1994 to provide structural organization and financial support to the municipal Family Health Teams (FHTs) [22]. FHTs are formed by one physician, one nurse, and four to six community health workers (CHW) to provide care for up to 4.000 patients in a given catchment area.

Despite the positive results in public health of the FHS [23–26], its expansion depended entirely on the municipalities' adherence to the policy. Rio de Janeiro, the last Brazilian capital to adopt the FHS, expanded the number of FHTs from 2008 to 2016, increased its coverage from 3.5% to 70% of the population [27], created new community-based primary care clinics and made strong investments to expand RTFM [28]. More than 600 FPs have graduated so far from the three FM residency programs established in the city, increasing the provision and fixation of FPs in PHC.

Today, 25% of the FHTs in Rio de Janeiro have trained FPs, 65% have physicians without RTFM (Generalists), and 10% have physicians enrolled at the More Doctors Program, a federal policy established in 2013 for provision and fixation of Brazilian and foreign physicians in PHC [29]. This distribution created a convenient quasi-experimental design to address the effect of RTFM in the provision of PHC.

### Outcomes

Three groups of outcomes were analyzed as dichotomous events: (1) detection of CHCs; (2) LTs requests; and (3) follow-up visits.

The risk of a patient being detected with a CHC was estimated for each condition considering only consultations among patients that have not had that specific condition diagnosed prior to the encounter. A list with 31 CHC was created combining the ICD-10 codes from three different frameworks: (a) the Brazilian list of ambulatory-care sensitive conditions [30], (b) the Charlson comorbidity index [31] and (c) the Elixhauser comorbidity index [32]. Chronic hepatitis was added to the list and Neoplastic diseases were divided into five subdomains: Cancer (general), Cancer in men (Neoplasia of male genital organs), Cancer in women (Neoplasia of female genital organs), Breast cancer (women only) and Metastatic cancer. Three conditions – Hypertension, Type 2 Diabetes Mellitus (T2DM), and Hypothyroidism – were categorized in two different ways: one using only ICD10 codes and the other using specific clinical criteria. Table 1 summarizes the list with the 31 CHCs, the respective ICD10 codes, and clinical criteria.

**Table 1:** List of chronic health conditions according to their ICD10 codes and clinical criteria

Hypertension (ICD10)	I10-I15
Hypertension clinical criteria	At least one measurement > 140/90 mmHg
Diabetes Mellitus (ICD10)	E10-E14
Diabetes clinical criteria	At least one glycemia > 126 mg/dl or A1C hemoglobin > 6.5%
Hypothyroidism coded	E00-E03, E890
Hypothyroidism clinical criteria	ICD codes (E00-E03, E890) and at least one TSH > 5 mcg/dL
HIV/AIDS	B20-B24, Z21, F024, R75
Drug addiction	F11-F16, F18, F19, Z715, Z722
Depression	F204, F313-F315, F32, F33, F341, F412, F432
Psychosis	F20, F22-F25, F28, F29, F302, F312, F315
Alcohol abuse	F10, E52, G621, I426, K292, K700, K703, K709, T51, Z502, Z714, Z721
Cardiac arrythmias	I441-I443, I456, I459, I47-I49, R001, Z950
Ischemic heart disease	I20-I22, I238, I248, I249, I250-I252, I255, I256, I258, I259
Peripheral artery disease	I70, I71, I731, I738, I739, I771, I790, I792, K551, K558, K559, Z958, Z959
Heart failure	I099, I110, I130, I132, I420, I425-I429, I43, I50, P290, J81
Kidney failure	I120, I131, N032-N037, N052-N057, N18, N19, N250, Z490-Z492, Z992
Osteoarthritis	M15- M19, M2, M40-M43
Rheumatic disorders	L93, L940, L941, L943, M05, M06, M08, M10, M11, M120, M123, M30, M310, M313, M315, M32-M36, M45, M461, M468, M469
Neurological disorders	G10-G13, G20-G22, G254, G255, G312, G318, G319, G32, G35-G37, G931, G934, G43, G44, G45, G46, G47, G5, G6, G7, G8, R47
Epilepsy	G40, G41, R56
Stroke	G45, G46, H340, I6
Dementia	F00-F03, F051, G30, G310, G311
COPD	I278, I279, J40-J44, J47, J60-J67, J684, J701, J703
Asthma	J45, J46
Chronic hepatitis	B180, B181, B182, B188, B189, K713, K714, K715, K730, K731, K732, K738, K739
Cirrhosis of the liver	B18, K700-K704, K709, K713, K715, K717, K73, K74, K760, K762-K764, K768, K769, Z944, I85, I864, I982, K711, K721, K729, K765-K767
Cancer (general)	C0, C1, C2, C3, C4, C64-C69, C7, C8, C9
Cancer in men	C60-C63
Cancer in women	C51-C58
Breast cancer	C50
Metastatic Cancer	C77, C78, C79, C80

For the risk of a LT being requested in one consultation, a list with the 30 most requested LTs in the sample was created. For LH, FSH, Rubella IgG and Rubella IgM, only women were considered as patients at risk, while PSA considered only men. For each LT in the list, consultations were categorized as having the LT requested (event) or not having the LT requested (no-event).

The risk of patients with a CHC having a follow-up consultation was estimated considering all patients seen in a month by each physician and consultations were clustered and ordered among each individual physician. Undiagnosed patients’ consultations were considered as no-event and diagnosed patients’ consultations were considered as events. The consultation in which the patient was diagnosed for the specific CHC was excluded.

The outcomes were chosen to approach three common questions that policymakers in LMIC have about the necessity of RTFM to work in PHC: “RTFM will make doctors more capable of detecting CHC?”, “Will they order less LTs?” and “Will they provide more follow-up visits? These are all common events in ambulatory care and analyzing the effect that RTFM has in their occurrence may bring relevant evidence for policymakers and health managers. The rationale behind the choice for these outcomes goes aligned with the definition of FM from the Brazilian [10], Canadian [20] and European [19] curricula for FM, i.e., that experts in FM are skilled clinicians that are capable of managing a full range of health conditions, making efficient use of diagnostic and therapeutic interventions.

In Rio de Janeiro, a comprehensive list of LTs is available for all doctors and nurses in the public PHC system to make use of. With no restriction for requesting any LT, the decision to order it or not will rely mostly on the doctor’s clinical judgment facing the patient’s health needs. In this scenario, concerns that patients would not have access to LTs due to poor availability and scarce resources moves from “lack of healthcare” to the “overuse that does not add value for patients and may even cause harm” [33]. Supported by several medical associations, campaigns such as Choosing Wisely [34] and the concept of Quaternary Prevention [35] try to engage physicians and patients in conversations about unnecessary tests, treatments and procedures, showing that “doing more does not mean doing better”. If RTFM helps doctors to make more efficient use of diagnostic tests, patients treated by FPs will probably have fewer LTs requested in their consultations.

### Exposures

Physicians were divided into two categories: (1) Generalists – doctors without RTFM (reference group); and (2) Family physicians (FPs) – graduated FPs, FM preceptors and residents enrolled in the FM residency programs.

Residents in FM spend two years working 48 hours a week in a community-based primary care clinic under the full supervision of a senior FP (FM preceptor) sharing responsibilities for the same patients in one FHT. Their clinical and academic [36, 37] activities aim to develop the skills, competencies, and attitudes a FP must have to practice in PHC. They are aligned with the National Committee for Medical Residencies (CNRM) [38] and with the Brazilian Society of Family and Community Medicine (SBMFC) [10]. Information about other forms of post-graduate training or specialization were not available in the database and were not taken into account, nor the number of years in practice for any doctor.

### Independent variables

Models were adjusted for information regarding the consultation context, such as patient’s age, patient’s Charlson Comorbidity Index [31] (except when the dependent variable was a CHC component of the index, e.g., HIV/AIDS, heart failure, stroke or COPD, since the same information would be present on both sides of the equation), and medical category in charge of the consultation (generalists or FPs).

The Charlson Comorbidity Index added to the models information about patients’ morbidity burden, assuming that those with a higher morbidity burden would be more likely to have more follow-up visits and to develop other CHCs, and would require more LTs. A dichotomous variable identifying if the consultation was a prenatal care visit was also included. As for time effects dummy variables for months and years were regarded in all models.

Patients’ information that does not change over time, such as patient’s sex and the Social Development Index (SDI) of the neighborhood were also considered. The SDI is a composite indicator combining information about sanitation, schooling, income, and housing conditions from every household in the FHT catchment area [39]. It represents the grade of social development of the neighborhood where the patient lives, varying from 0 (least developed) to 1 (most developed).

All clinics and FHTs in this sample have the same human resources, equipment, and physical structure. Generalists and FPs were evenly distributed across the neighborhoods, clinics, and FHTs.

### Statistical analyses

Multilevel binomial regression models were built to estimate the relative risks between Generalists (reference group) and FPs of each one of the three types of outcomes happening in medical consultations in primary care.

For LTs requested in one consultation and the detection of CHCs, consultations were clustered and ordered per each individual patient. This created a hierarchical data structure in which consultations were nested within patients, hence taking into account the correlation among consultations from the same patient. These models were adjusted for first level covariates (consultation), i.e., patient’s age, patient’s Charlson Comorbidity Index, prenatal care consultation, time, and medical category; and for second level covariates – SDI and patient’s sex.

For follow-up consultations, the hierarchical data structure chosen had consultations (first level) nested within doctors (second level). This structure captures the availability of consultations (access to healthcare) for each CHC between Generalists and FPs. In this way, RRs represent the risk of FPs offering one consultation for a specific CHC in a given period, compared to generalists. These models were adjusted for first level covariates (consultation), i.e., patient’s age, patient’s Charlson Comorbidity Index, prenatal care consultation time, SDI and patient’s sex; and second level covariates (medical category).

With the RRs from the previous models, Population Attributable Fractions (PAFs) for each LT and CHC were calculated to estimate the change in the number of LTs requested and in the number of incident cases of CHCs per year in the same health care district if all medical consultations were performed by trained FPs [40, 41]. Data processing and statistical analysis were performed using R version 3.6.2 and lme4 package.

## Results

Over a period of 6 years, 569.289 patients (335.346 women and 233.943 men) had 2.908.864 medical consultations performed by 964 different doctors (734 generalists and 231 FPs) in 30 PHC clinics and 196 FHTs. Doctors worked non-concurrently throughout the period of analysis and generalists accounted for 66.4% of the consultations in the sample, while FPs were responsible for 33.6%. There was a small but statistically significant (p-value for t-test < 0.05) difference in patients’ age and SDI distributions among Generalists’ and FPs’ populations. Patients from the wealthiest (SDI = 0.689) and the poorest areas (SDI = 0.416) were seen by both categories (Table 2).

**Table 2:** Number of medical consultations and patients’ characteristics according to each medical category in the study sample. Rio de Janeiro, Brazil, 2015 – 2018

Medical category	Number of doctors N (%)	Consultations N (%)	SDI mean (SD)	Patients age mean (SD)	Consultations according to sex – N (%)	
					Women	Men
Generalists	734 (76.1)	1.932.297 (66.4)	0.573 (0.03)	40.3 (24.0)	1.267.756 (65.6)	664.541 (34.4)
Family physicians	231 (23.9)	976.567 (33.6)	0.581 (0.03)	40.71 (23.1)	645.175 (66.1)	331.392 (33.1)

Hypertension, T2DM, and Hypothyroidism, the three CHCs that were categorized using both ICD10 and clinical criteria, presented a similar pattern: using ICD10 codes, FPs were less likely to diagnose these conditions, while using clinical criteria, FPs were more likely to diagnose all of them.

FPs were more likely to diagnose the majority of the remaining 24 domains of CHC in the list. The exceptions were Epilepsy, presenting a lower risk of being diagnosed by FPs, while Rheumatic disorders, Cirrhosis of the liver and Neoplasia of male genital organs presented similar risk between generalists and FPs. Metastatic cancer presented a higher but not statistically significant risk of being diagnosed by FPs.

The risk of a patient having a follow-up visit was higher for FPs running the consultation. Even for those patients that have CHC that FPs were less likely to diagnose, patients have a higher chance of having a follow-up visit if their doctor is a FP. Around 7% of the patients coded as having Hypertension or T2DM would no longer be coded as such, decreasing by 1208, and 384 the number of incident cases per year, respectively. On the other hand, in all domains, except those that RR for medical categories were not statistically significant, an increasing number of incident cases would be seen. Hypertension and T2DM (according to clinical criteria), Heart failure, Stroke, Asthma, and Osteoarthritis would have the biggest increment in the number of incident cases, while Drug addiction, Alcohol abuse, Kidney failure, and Peripheral artery disease would have the biggest relative increase of new cases (Table 3).

**Table 3:** Relative risks (RR) and 95% confidence intervals for a patient being diagnosed for a chronic health condition in one medical consultation and RRs for follow; up consultations happening in a month in PHC between Generalists (reference) and Family physicians. Rio de Janeiro, Brazil, 2013- 2018

	Family physicians	
Condition	Detection of CHCs	Follow-up
Hypertension - ICD10	0.84 (0.82-0.86)	1.18 (1.10-1.27)
Hypertension - clinical criteria	1.15 (1.13-1.17)	1.54 (1.41-1.68)
Diabetes Mellitus - ICD10	0.81 (0.79-0.84)	1.14 (1.09-1.20)
Diabetes Mellitus - clinical criteria	1.07 (1.04-1.11)	1.43 (1.32-1.55)
Hypothyroidism - ICD10‡	0.97 (0.91-1.04)	1.60 (1.45-1.77)
Hypothyroidism - clinical criteria	1.51 (1.25-1.82)	2.25 (1.86-2.72)
AIDS	1.19 (1.09-1.29)	1.72 (1.55-1.91)
Drug addiction	1.98 (1.76-2.22)	2.64 (2.22-3.14)
Alcohol abuse	1.72 (1.54-1.91)	2.53 (2.13-2.99)
Depression	1.19 (1.14-1.26)	1.89 (1.70-2.11)
Psychosis	1.20 (1.11-1.30)	1.77 (1.58-1.98)
Cardiac arrhythmias	1.31 (1.19-1.45)	1.93 (1.70-2.19)
Peripheral artery disease	2.41 (2.05-2.82)	2.80 (2.26-3.47)
Ischemic heart disease	1.38 (1.27-1.49)	2.01 (1.77-2.27)
Heart failure	1.69 (1.56-1.83)	2.66 (2.34-3.01)
Kidney failure	1.82 (1.64-2.02)	2.44 (2.08-2.87)
Osteoarthritis	1.11 (1.07-1.15)	1.77 (1.57-1.98)
Rheumatic disorders‡	1.00 (0.92-1.10)	1.74 (1.56-1.93)
Neurological disorders	1.29 (1.24-1.34)	1.94 (1.75-2.15)
Epilepsy	0.86 (0.79-0.94)	1.37 (1.24-1.51)
Stroke	1.44 (1.34-1.55)	1.73 (1.55-1.94)
Dementia	1.28 (1.16-1.42)	1.78 (1.56-2.05)
COPD	1.11 (1.02-1.21)	1.56 (1.41-1.72)
Asthma	1.28 (1.21-1.36)	1.71 (1.51-1.93)
Chronic hepatitis	1.31 (1.10-1.57)	1.85 (1.59-2.15)
Cirrhosis of the liver‡	1.03 (0.90-1.18)	1.99 (1.77-2.24)
Cancer	1.26 (1.17-1.36)	1.78 (1.43-2.20)
Neoplasia - Men‡	1.11 (0.92-1.33)	2.14 (1.75-2.62)
Neoplasia - Women	1.34 (1.11-1.62)	2.08 (1.77-2.46)
Breast cancer	1.42 (1.21-1.67)	1.69 (1.06-2.70)
Metastatic cancer‡	1.47 (0.96-2.26)	1.18 (1.10-1.27)

‡: Non-statistically significant in the multivariate binomial models

All p-values were smaller than 0.001, except for Neoplasia – Women (p-value < 0.005) and non-statistically significant for those marked with ‡

Multilevel binomial regression models adjusted by patients’ age, sex, time, Charlson comorbidity index, and SDI

Generalists (reference group) are doctors without residency training in Family Medicine

Family physicians are graduated FPs, FM preceptors and residents enrolled in Family Medicine training

Patients seen by FPs present a lower risk of having any of the 30 LTs requested during one consultation, with a RR lower than 50% for Glucose, Ova & parasite, Urea, Sodium, T3, T4, PSA Rubella IgG and IgM, Uric acid, Calcium, AST, ALT and LDL cholesterol (Table 4).

**Table 4:** Population Attributable Fractions (PAFs) and the change in the absolute numbers of chronic health conditions detected per year in a scenario in which all medical consultations were performed by Family Physicians. Rio de Janeiro, 2013- 2018

Condition	Population attributable fraction	Cases per year	Number of cases added or subtracted
Hypertension - ICD10	-11.2 (-9.8; -12.7)	15901	-1781 (-1558; -2019)
Hypertension - clinical criteria	9.5 (8.3; 10.7)	11681	1110 (970; 1250)
Diabetes Mellitus - ICD10	-13.5 (-11.2; -15)	5562	-751 (-623; -834)
Diabetes Mellitus - clinical criteria	4.5 (2.6; 7)	4089	184 (106; 286)
Hypothyroidism - ICD10^a^	-2 (-6.2; 2.6)	755	-15 (-47; 20)
Hypothyroidism - clinical criteria	28.9 (15.3; 42.7)	79	23 (12; 34)
AIDS	11.9 (5.8; 17.6)	415	49 (24; 73)
Drug addiction	49 (40.2; 57.5)	197	97 (79; 113)
Alcohol abuse	38.5 (30.4; 46.3)	237	91 (72; 110)
Depression	11.9 (8.9; 15.9)	1119	133 (100; 178)
Psychosis	12.4 (7; 18.1)	466	58 (33; 84)
Cardiac arrhythmias	18.7 (11.9; 26)	412	77 (49; 107)
Peripheral artery disease	63.6 (51.6; 75)	114	73 (59; 86)
Ischemic heart disease	22.4 (16.4; 28)	499	112 (82; 140)
Heart failure	37.2 (31.3; 43.1)	482	179 (151; 208)
Kidney failure	42.7 (35; 50.5)	270	115 (94; 136)
Osteoarthritis	7 (4.5; 9.5)	3800	266 (171; 361)
Rheumatic disorders^a^	0 (-5.5; 6.4)	503	0 (-28; 32)
Neurological disorders	17.6 (14.8; 20.3)	2092	368 (310; 425)
Epilepsy	-9.8 (-4.1; -15)	579	-57 (-24; -87)
Stroke	25.5 (20.3; 30.8)	586	149 (119; 180)
Dementia	17 (10.1; 24.5)	310	53 (31; 76)
COPD	7 (1.3; 13)	532	37 (7; 69)
Asthma	17 (13; 21.3)	782	133 (102; 167)
Chronic hepatitis	18.7 (6.4; 31.8)	84	16 (5; 27)
Cirrhosis of the liver^a^	2 (-6.9; 11.3)	212	4 (-15; 24)
Cancer	15.9 (10.7; 21.3)	627	100 (67; 134)
Neoplasia - Men^a^	7 (-5.5; 19.7)	92	6 (-5; 18)
Neoplasia - Women	20.3 (7; 34.1)	81	16 (6; 28)
Breast cancer	24.5 (13; 36.3)	122	30 (16; 44)
Metastatic cancer^a^	27 (-2.7; 58.8)	16	4 (0; 9)

^a^: Non-statistically significant in the multilevel multivariate binomial models

In a scenario where all medical consultations would be performed by FPs, T3, T4, Ova & parasite, Uric acid, PSA, Urea and LDL cholesterol would have their demand reduced by more than 50%. In absolute numbers, Hemograms, Urea, Uric acid, LDL cholesterol and Urinalysis would experience the greater impact, with almost 20.000 Hemograms and more than 10.000 Urea, Uric acid, and LDL cholesterol tests avoided per year (Table 5)

**Table 5:** Relative risks and 95% confidence intervals for a laboratory test (LT) being requested in one medical consultation comparing Family physicians and Generalists (adjusted for age, sex, social development index and Charlson comorbidity index) in PHC, the respective Population Attributable Fractions (PAFs) and the change in the absolute numbers of LTs requested in a scenario in which all medical consultations were performed by Family Physicians. Rio de Janeiro, 2013- 2018

Laboratory test	Family Physicians (95% CI)	PAF (95% CI)	Absolute number of LTs requested per year	Absolute change in the total number of LTs requested per year (95% CI)
Hemogram	0.53 (0.53-0.54)	-37.1 (-36.1; -37.1)	51757	-19202 (-18684; -19202)
Creatinine	0.85 (0.84-0.86)	-10.5 (-9.8; -11.2)	43746	-4593 (-4287; -4900)
Urea	0.29 (0.28-0.29)	-61.9 (-61.9; -63.1)	22577	-13975 (-13975; -14246)
Sodium	0.42 (0.41-0.43)	-47.8 (-46.8; -48.9)	10517	-5027 (-4922; -5143)
Potassium	0.73 (0.72-0.75)	-19.7 (-18.1; -20.5)	18265	-3598 (-3306; -3744)
Glucose	0.46 (0.45-0.47)	-43.8 (-42.8; -44.8)	9072	-3974 (-3883; -4064)
A1C Hemoglobin	0.64 (0.63-0.65)	-27.2 (-26.3; -28.1)	25720	-6996 (-6764; -7227)
Total cholesterol	0.81 (0.8-0.82)	-13.5 (-12.7; -14.2)	34673	-4681 (-4403; -4924)
HDL cholesterol	0.94 (0.93-0.95)	-4.1 (-3.4; -4.8)	31127	-1276 (-1058; -1494)
LDL cholesterol	0.41 (0.41-0.42)	-48.9 (-47.8; -48.9)	22163	-10838 (-10594; -10838)
Triglycerides	0.82 (0.81-0.83)	-12.7 (-12; -13.5)	33828	-4296 (-4059; -4567)
Uric acid	0.23 (0.22-0.23)	-69 (-69; -70.2)	15603	-10766 (-10766; -10953)
TSH	0.71 (0.7-0.73)	-21.3 (-19.7; -22.2)	11679	-2488 (-2301; -2593)
Triiodothyronine	0.08 (0.06-0.09)	-88.4 (-87; -91.2)	791	-699 (-688; -721)
Thyroxine	0.13 (0.12-0.15)	-81.6 (-79; -83)	886	-723 (-700; -735)
free Thyroxine	0.56 (0.55-0.58)	-34.3 (-32.5; -35.2)	5432	-1863 (-1765; -1912)
Bilirubin	0.78 (0.75-0.81)	-15.8 (-13.5; -18.1)	2304	-364 (-311; -417)
AST	0.48 (0.47-0.49)	-41.8 (-40.9; -42.8)	9181	-3838 (-3755; -3929)
ALT	0.47 (0.46-0.48)	-42.8 (-41.8; -43.8)	9363	-4007 (-3914; -4101)
Alkaline phosphatase	0.69 (0.67-0.71)	-23 (-21.3; -24.7)	3505	-806 (-747; -866)
Gamma-GT	0.64 (0.62-0.66)	-27.2 (-25.5; -28.9)	4386	-1193 (-1118; -1268)
ESR	0.74 (0.71-0.77)	-18.9 (-16.6; -21.3)	1957	-370 (-325; -417)
Ova & parasite	0.09 (0.08-0.11)	-87 (-84.3; -88.4)	604	-525 (-509; -534)
Urinalysis	0.67 (0.67-0.68)	-24.7 (-23.8; -24.7)	41333	-10209 (-9837; -10209)
Calcium	0.44 (0.42-0.46)	-45.8 (-43.8; -47.8)	1866	-855 (-817; -892)
LH	0.56 (0.52-0.61)	-34.3 (-29.8; -38)	674	-231 (-201; -256)
FSH	0.6 (0.56-0.64)	-30.7 (-27.2; -34.3)	863	-265 (-235; -296)
Rubella IgG	0.48 (0.41-0.55)	-41.8 (-35.2; -48.9)	210	-88 (-74; -103)
Rubella IgM	0.47 (0.4-0.54)	-42.8 (-36.1; -49.9)	216	-92 (-78; -108)
PSA	0.36 (0.34-0.37)	-54.1 (-53.1; -56.3)	4365	-2361 (-2318; -2457)

All p-values were smaller than 0.001

Generalists (reference group) are doctors without residency training in Family Medicine

Family physicians are graduated FPs, FM preceptors and residents enrolled in Family Medicine training

**Fig. 1:**
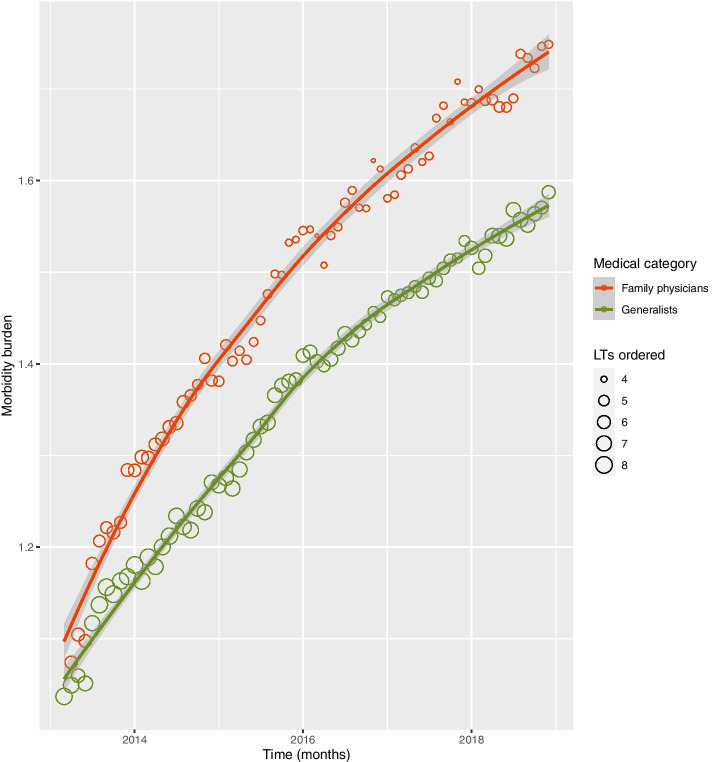
Average morbidity burden of adult patients seen in PHC and average amount of laboratory tests (LTs) requested in one consultation according to the medical category of the health care provider from January 2013 to December 2018, in Rio de Janeiro, Brazil

Figure 1 presents a summary picture of patients with at least one CHC seen in PHC by FPs and Generalists throughout the analysis. Each dot represents, for each category in a month, the average amount of LTs ordered (size) and the average amount of CHC that patients have (y-axis). They don’t represent reasons for encounter, but the number of morbidities that patients have listed in their records. For Hypertension, T2DM, and Hypothyroidism only patients that met clinical criteria were considered. Over the years, the amount of CHC increase for both groups of patients, but that increment is bigger for FPs’ patients, that also have fewer LTs requested.

## Discussion

The hypothesis tested here show that RTFM, when compared to having no training in FM, can make doctors more likely to diagnose a large set of CHC, provide more follow-up visits to their patients and request fewer LTs in primary care. Moreover, it seems that RTFM makes doctors more accurate when diagnosing Hypertension, T2DM and Hypothyroidism, the three domains analyzed using ICD10 codes and clinical criteria.

These findings support the definition of FPs as skilled clinicians able to cover the “full range of health conditions” and to use “the prevalence and incidence of an illness in the community” during their decision-making processes [10, 11, 20]. This is important evidence showing that FM is a key element to bring comprehensiveness to PHC. Being more capable of detecting a large set of CHC together with the increased offer of follow-up visits to patients with CHCs means not only that more patients will have their health issue identified but that they will also have more medical visits to manage their CHC, which is key for the success of the treatment.

Furthermore, the higher risk of patients being diagnosed for a CHC by FPs can be representing the detection of conditions when symptoms are mild or non-specific, in the early stages [9, 42]. If patients with undifferentiated symptoms leave the consultation room without a diagnosis – or a clear diagnostic plan – they will potentially go to another health service to find a solution to their problem. Their diagnosis will be delayed, and it will probably be made when the symptoms become more pronounced. For conditions such as Cancer, HIV, Cardiac arrhythmias, and Kidney failure this delay can make a difference between a successful treatment and an avoidable death. The changes in the absolute numbers in Table 3 does not mean that those patients are not being diagnosed, but they are either trying to solve their problems somewhere else or having their diagnosis delayed.

The disparity of RRs observed in the three domains analyzed using ICD10 codes and clinical criteria – Hypertension, T2DM and Hypothyroidism – also brings to discussion the matter of payment-for-performance schemes, overdiagnosis and accuracy.

Hypertension and T2DM are CHCs that are targeted by the payment-for-performance municipal schemes since the PHC reform took place [27]. One performance measurement is the number of patients coded for Hypertension or T2DM reaching levels of blood pressure below 140/90 mmHg or 6.5% for A1C hemoglobin. If patients with prediabetes or prehypertension are coded as T2DM and Hypertension, their control target would be already reached, making these patients accounted as successfully treated. Another reason for that difference could be the focus that the federal government gives to cardiovascular diseases, via selective programs for screening and management of Hypertension and T2DM. With financial and political incentives for FHTs to pay extra attention to these conditions, it is not unusual for FHTs to reserve specific days or shifts of the week in their schedule exclusively for patients with Hypertension and/or T2DM, restricting access for patients with other health conditions [43, 44].

Payment-for-performance initiatives are designed to improve the quality of care, but if it comes with an increased risk of overdiagnosis [45], the potential damage that can result from that (overtreatment, unnecessary procedures, and stigmatization) can be more harmful than beneficial [46]. Furthermore, a higher number of Hypertension and T2DM cases should be followed by a proportionally increased number of actual cardiovascular diseases (Heart failure, Ischemic heart disease, Stroke, Cardiac arrhythmias, and Peripheral artery disease), but that is not the case.

One could argue that those patients were not subject to overdiagnosis, but were actual cases that were properly managed and, therefore, have the development of cardiovascular diseases prevented. That could be true for cardiovascular diseases, however, the increased risk that FP’s patients have to be diagnosed for HIV, Dementia, Drug addiction, Psychosis, Kidney failure, Cardiac arrhythmias, and Cancer suggests the opposite. These are conditions that need not only good clinical knowledge and skills to be suspected and diagnosed but competencies and attitudes that RTFM programs are meant to develop [10, 19, 20, 47].

The higher risk of detection of HIV among FPs illustrates this situation. Rapid tests for HIV and syphilis are available in every public PHC clinic in Rio de Janeiro. Doctors, nurses, and nurse technicians are trained by the RJ-MHD to perform this point-of-care test. They learn the procedure but raising suspicion and offering the test to a patient demands attitudes that are beyond the knowledge and skills needed to perform the test.

Overdiagnosis can be also the case for Epilepsy, a CHC referring to recurrent seizures, but patients with isolated seizures or even febrile seizures are commonly labeled as epileptic.

Mental health conditions (Depression, Psychosis, Drug addiction and Alcohol abuse) being more likely to be diagnosed by FPs also show how RTFM can make doctors more sensitive to perceiving conditions that are commonly neglected in the community, which require an active attitude to be suspected, investigated and diagnosed. In other words, if the professional just waits for the patient to declare having problems with alcohol or drugs, very few patients will be diagnosed and will have an opportunity for treatment [48].

It is worth noting the contrast between the amount of LTs requested and the number of CHCs diagnosed, e.g., FPs request 64% less PSA than Generalists, but the detection of Cancer in male genital organs (93% are Prostate Cancer) is similar. The number of glucose and A1C hemoglobin exams is 30 to 50% higher among Generalists, but the number of patients diagnosed by clinical criteria for T2DM and the risk of follow-up consultations favors FPs. This information corroborates the definition of a FP as a “skilled clinician” that makes “effective and efficient use of diagnostic and therapeutic interventions” [19, 20]. LTs that would usually be requested for patients with liver damage, i.e., Gamma GT, AST, ALT, and Alkaline phosphatase illustrates the situation very well. They are more often requested by Generalists but conditions that would necessarily need those exams, such as Alcohol abuse and Chronic hepatitis, are more likely to be diagnosed by FPs. Conditions that would also require those exams, such as Cholangitis and Cirrhosis of the liver, could be partially accountable for that difference, but they would hardly explain the whole gap.

Finally, continuity of care can also play an important role in increasing the risk of detection of CHC among FPs. Since FPs provide more follow-up consultations, their patients will have more opportunities to be diagnosed for a second or third CHC, a common event in patients with multimorbidity [49]. Cardiac arrhythmias, Kidney failure and Peripheral artery disease, conditions that are consequences of having a prior diagnose of T2DM or Hypertension, exemplify this phenomenon.

### Strengths and limitations

The decision to address the subject by approaching policymakers’ and managers’ general questions about the effect of RTFM in healthcare limits our conclusions to the evidence that two years of training increases the risk of CHCs being detected, of follow-up visits being offered and lowers the risk of LTs being requested in medical consultations in primary care. To infer that RTFM allows doctors to provide better health care in every aspect of medical practice based solely on the results from our study would be a disproportionate and unfair generalization. To conclude that RTFM influences other types of outcomes, such as lowering mortality, decreasing hospital admissions, and promoting quality of life, different approaches and research methods must be taken.

However, using real-world data from a real experience in a middle-income setting shows us evidence about the real performance of physicians in primary care and how it can be affected by two years of training in FM, making it more likely that the implementation of this type of a training program in other scenarios will produce similar results.

## Conclusion

Being a competent clinician, able to cover the full range of health conditions and making effective use of diagnostic and therapeutic interventions are competencies that FPs must have to work in PHC, and RTFM seems to be effective in promoting them among physicians in Rio de Janeiro. Furthermore, the development of these competencies helps to expand access to health care, making PHC more comprehensive, effective, and socially accountable. Policymakers in LMIC must consider that PHC systems will not move from selective interventions to become truly comprehensive and effective without investments in capacity building of human resources. Regarding the medical workforce, this transformation will not happen without investments in RTFM.

## Data Availability

All data used in this research represent patients, health care providers and medical consultations information that are under the protection of the Rio de Janeiro Municipal Health Department. This data can be obtained from the Rio de Janeiro Superintendence of Primary Care (sapsmsrj@gmail.com) under the authorization of the Rio de Janeiro Municipal Health Department Research Ethics Committee (cepsmsrj@yahoo.com.br).
